# Human mobility and the infectious disease transmission: A systematic review

**DOI:** 10.1080/10095020.2023.2275619

**Published:** 2023-11-29

**Authors:** M. Naser Lessani, Zhenlong Li, Fengrui Jing, Shan Qiao, Jiajia Zhang, Bankole Olatosi, Xiaoming Li

**Affiliations:** a Geoinformation and Big Data Research Lab, Department of Geography, University of South Carolina, Columbia, USA; b Big Data Health Science Center, University of South Carolina, Columbia, USA; c Department of Health Promotion, Education, and Behavior, University of South Carolina, Columbia, USA; d Department of Epidemiology and Biostatistics, Arnold School of Public Health, University of South Carolina, Columbia, USA; e Department of Health Services Policy and Management, Arnold School of Public Health, University of South Carolina, Columbia, USA

**Keywords:** Human mobility, geography, infectious diseases, sexually transmitted diseases, systematic review

## Abstract

Recent decades have witnessed several infectious disease outbreaks, including the coronavirus disease (COVID-19) pandemic, which had catastrophic impacts on societies around the globe. At the same time, the twenty-first century has experienced an unprecedented era of technological development and demographic changes: exploding population growth, increased airline flights, and increased rural-to-urban migration, with an estimated 281 million international migrants worldwide in 2020, despite COVID-19 movement restrictions. In this review, we synthesized 195 research articles that examined the association between human movement and infectious disease outbreaks to understand the extent to which human mobility has increased the risk of infectious disease outbreaks. This article covers eight infectious diseases, ranging from respiratory illnesses to sexually transmitted and vector-borne diseases. The review revealed a strong association between human mobility and infectious disease spread, particularly strong for respiratory illnesses like COVID-19 and Influenza. Despite significant research into the relationship between infectious diseases and human mobility, four knowledge gaps were identified based on reviewed literature in this study: 1) although some studies have used big data in investigating infectious diseases, the efforts are limited (with the exception of COVID-19 disease), 2) while some research has explored the use of multiple data sources, there has been limited focus on fully integrating these data into comprehensive analyses, 3) limited research on the global impact of mobility on the spread of infectious disease with most studies focusing on local or regional outbreaks, and 4) lack of standardization in the methodology for measuring the impacts of human mobility on infectious disease spread. By tackling the recognized knowledge gaps and adopting holistic, interdisciplinary methods, forthcoming research has the potential to substantially enhance our comprehension of the intricate interplay between human mobility and infectious diseases.

## 1. Introduction

Human movement, as a critical component of human contact and connectivity, is one of key drivers of the infectious diseases transmissions, particularly during a pandemic. Human mobility is widely defined as individuals’ or groups’ movement in space and time, including displacement, rural to urban, household fluidity, domestic and international migrations, and involuntary mobility like human trafficking for sexual exploitation, migration associated with armed conflicts, and forced labor. With the increase of individual and collective human movement due to globalization and massive transportation networks, the spread of infectious diseases has also grown on an unprecedented scale, consequently leading to an epidemic or a pandemic. Humans have witnessed several pandemics throughout history. For example, Black Death in the 14^th^ Century is believed to have triggered quarantine in Europe. Despite the undeveloped state of transportation systems, mobility was considered a key element in the widespread transmission of the Black Death (Duncan and Scott 2005). The 1918 Spanish influenza infected hundreds of millions and led to the death of approximately fifty million people (Johnson and Juergen 2002); the 1968 flu pandemic, also known as Hong Kong flu that originated in China and lasted till 1970, is estimated to have caused between 0.5 to 2 million deaths worldwide (Saunders-Hastings and Krewski 2016). The 2009 HIN1 outbreak emerged in Mexico, and approximately simultaneous outbreaks started in Southern United States (Saunders-Hastings and Krewski 2016). In December 2019, a pneumonia was reported in Wuhan, China (Zhu et al. 2020), caused by a new type of coronavirus named SARS-CoV-2. To date, over 663 million confirmed cases and almost 6.72 million deaths have been reported worldwide. During these pandemics, various responses targeted restricting the spread of the virus. In the COVID-19 crisis, human mobility has been regarded as one of the major drivers of outbreaks worldwide. To curb human movement, a large number of countries instituted full or partial lockdown ranging from days to months, including domestic travel bans, international travel restriction, border closure, stay-at-home orders, prohibited public gatherings, as well as implementing closures of educational institutions, public transportation systems, and workplaces (Kharel et al. 2022, Barbieri et al. 2021).

Throughout various stages of pandemics, researchers have devoted significant resources to explore the relationship between infectious diseases and human mobility, seeking to uncover the patterns that link movement and virus spread. They have utilized an extensive array of simulation, mathematical, and prediction models on a diverse set of datasets. The use of human movement models to predict the spread of viruses has demonstrated that human mobility plays a crucial role in the transmission of infectious diseases, particularly during COVID-19 (Lee et al. 2023, Perrotta et al. 2022). Analogously, numerous models have been proposed for the transmission of Human Immunodeficiency Virus (HIV) (Saravanakumar et al. 2020), Ebola virus (Li 2017), and vector-borne diseases such as malaria persistence (Gomes et al. 2020), dengue cases (Heidrich et al. 2021) and Zika virus (Perrotta et al. 2022) among others. These studies consistently show that human movement plays a critical role in the transmission of infectious diseases. However, it is crucial to specify that mobility restrictions have shown to be effective primarily in the early stages of an epidemic when there is no available effective vaccine or medication. For instance, movement restrictions have been successful in slowing down transmission rates, as observed during the COVID-19 pandemic, they have not been sufficient in halting the spread entirely. On the other hand, such measures come with considerable economic, social, and psychological costs, particularly affecting marginalized communities (Roques et al. 2020, Panda et al. 2021, Bonal and Gonzalez 2020).

In this review, the authors comprehensively examined literature from the past two decades that focuses on the interplay between human mobility and the spread of infectious diseases. Specifically, our investigation is structured along four key dimensions to provide a coherent analysis of existing research. Firstly, we investigated the types of data sources predominantly employed in these studies. We evaluated the extent to which various data types, such as medical records, travel statistics, or social media feeds, have been harnessed to explore the relationship between human movement and disease transmission. Secondly, we turned our attention to methodological approaches, highlighting the range of techniques used in the literature. This includes an in-depth look at mathematical models, regression analyses, and predictive algorithms, among other methods, to assess their effectiveness and limitations in capturing the complexities of disease spread related to human mobility. Thirdly, we examined the scalability of the studies, categorizing them based on their geographical focus. This segmentation allows us to assess the breadth and limitations of existing research, particularly in terms of its applicability to broader contexts. Lastly, we focused on the degree of methodological standardization across studies. We assessed whether uniform approaches are used, and if not, how these variances impact the reliability and comparability of research findings. Based on these results, we synthesized and discussed the association between human mobility and infectious diseases. We then identified key areas for improvement in the field of public health and infectious diseases, including underutilized data sources and methodological diversity. By offering a roadmap for scalable research and advocating for methodological standardization, we provide actionable insights that build upon and refine earlier studies, ultimately aiming to enhance public health interventions and open new avenue of research for future studies.

To achieve these objectives, we searched relevant literature by querying human mobility and the chosen infectious diseases in two databases (Web of Science & PubMed). Web of Science is a comprehensive, multidisciplinary database that covers numerous subject areas and provides access to a vast array of high-quality, peer-reviewed articles, with PubMed specializing in life sciences and biomedical literature, ensuring a comprehensive and in-depth review of health research topics (Kulkarni et al. 2009, Sayers et al. 2023).

## 2. Methodology

### 2.1. Search strategy and eligibility criteria

The authors, experts in the fields of geography and public health, meticulously devised a search protocol. Furthermore, the review article adheres to the guidelines prescribed by the Preferred Reporting Items for Systematic Reviews and Meta-Analyses (PRISMA) (Moher et al. 2009). To identify relevant articles, two databases were systematically reviewed: Web of Science (WOS) Core Collection and PubMed. Both WOS and PubMed are reputable databases with a high degree of credibility and reliability, providing access to a wide range of peer-reviewed journals. These databases are thoroughly curated, and their content is typically more rigorous and reliable than other sources. WOS is a paid-access platform that enables researchers to access multiple databases in various academic disciplines from more than 1.9 billion cited references, mainly focusing on medicine and natural science (Ellegaard and Wallin 2015). PubMed, however, is an unpaid search engine allowing primarily over 35 million citations in the field of biomedical literature (Ossom Williamson and Minter 2019). Following the research protocol, we designed, we successfully retrieved a substantial number of papers from the two databases, which adequately support this review article.

The search terms used for downloading articles included “human mobility”, “infectious diseases”, and their synonyms. The keywords for “human mobility” and “infectious diseases” were collected based on previous studies (Jing et al. 2023) because no official lists exist for these two terms that we could refer to. Furthermore, Boolean operators, hyphens, and truncations were utilized to make the search as comprehensive as possible. To further broaden the coverage of the review, the references of reviewed articles were searched for additional literature. Ultimately, sources that clearly discussed the association between human mobility and infectious diseases were retained.

Articles were chosen when they were (1) peer-reviewed original research, (2) published in English journals, (3) quantitively assessed the association between human mobility and selected infectious diseases, and (4) published between January 2000 and December 2022, as the twenty-first century has been characterized by rapid technological and demographic changes, increased global connectivity, and urbanization, which coincide with a surge in infectious disease outbreaks. These articles reported observational studies, including cohort, cross-sectional, and models for prediction, to examine the association between human mobility and infectious diseases. Articles were excluded when they (1) were literature reviews, (2) conference proceedings, and (3) related to vaccination.

### 2.2. Screening and information extraction

The initial search obtained 2458 articles from the selected databases; of these results, 2129 articles were selected after eliminating duplicates. Since the initial search could include irrelevant articles based on the appearance of search terms in titles, abstracts, or content, a combination of unsupervised machine learning and manual screening was applied to identify relevant articles from the initial search pool.

First, the Lbl2Vec algorithm, an unsupervised document classification and retrieval method, was used to select literature that appeared relevant to the topic of interest based on titles and abstracts (Schopf, Daniel, and Florian 2022). The algorithm automatically produces jointly embedded labels, word vectors, and documents and then returns categories of documents based on predefined keywords. Once the representation of input documents and words is generated in a shared vector-space, the algorithm learns label vectors from predefined keywords, representing varieties of categories. Finally, the algorithm predicts the connection of documents to each category based on the similarity of documents vector with generated label vectors. For calculating the centroid $\overset{-}{e}$ of embedding, the below equation is used, where n represents the number of words.


(1) $$\overset{-}{e}=\frac{1}{n}\sum_{x=1}^{n}e_{x}$$

**Figure 1.**
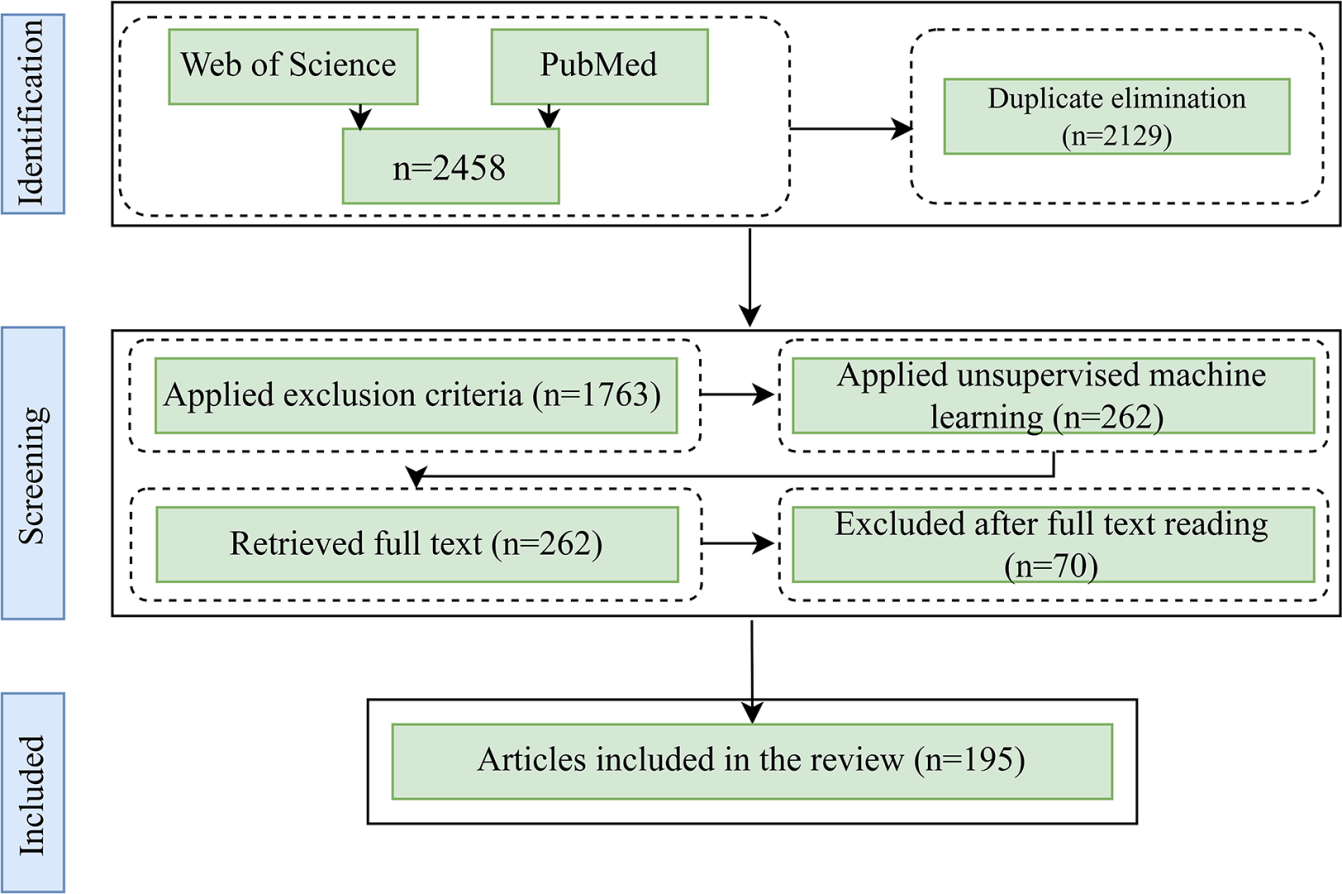
The flow diagram illustrates the process of article identification, screening, and selection. While the diagram shows the selection of 192 articles, three additional papers was manually included after examining 500 random articles from the unselected pool via unsupervised machine learning, bringing the total to 195.

Two hundred sixty-two articles were obtained after implementing the algorithm, and of these results, 192 distinctive papers were finalized after the manual screening. In addition, to ensure that the algorithm detected relevant articles, the authors randomly selected 500 papers with a higher score of similarity from unselected publications. After reading their titles and abstracts, only three of them was deemed relevant and included in the review. Next, full-text versions were obtained for those articles that were recognized to be relevant. Following this, the articles were summarized, and information was extracted pertaining to the study purpose, data types, methodological approaches, country, and main findings. In this review, the focus was solely on the relationship between human mobility and the selected infectious diseases and the relevant outcomes, even though some studies may have explored additional relationships. Figure 1 illustrates the article identification and selection process.

## 3. Result

### 3.1. Overview

**Figure 2.**
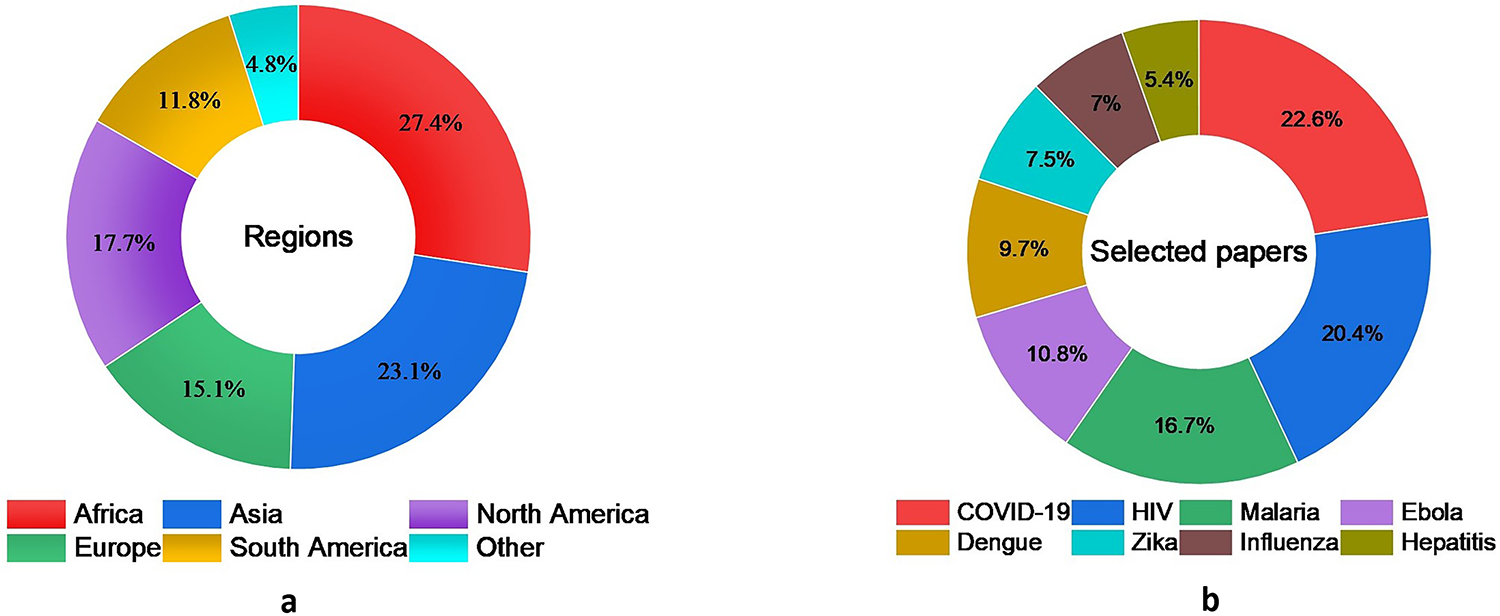
A total of 195 articles were selected for review: the distribution of these articles by disease type is presented in (a); (b) visualizes the percentage of selected studies by region, providing insights into the geographic focus of research on infectious diseases in this review.

The number of articles explicitly discussed the association between human mobility and infectious diseases varied based on the type of disease. Some infectious diseases, for example, have gained more attention compared to others. Since there are more than 27 infectious diseases based on (Jing et al. 2023), discussing human mobility and all of these diseases is beyond the scope of this paper. Consequently, this review paper only includes infectious diseases for which we were able to retrieve more than ten relevant articles from WoS and PubMed databases using our search terms. These articles must have clearly discussed the connection between human mobility and the transmission of infectious diseases. The final included diseases are vector-borne illnesses (Zika virus, dengue and malaria), Ebola, HIV, influenza, hepatitis, and COVID-19, as presented in Figure 2.

**Figure 3.**
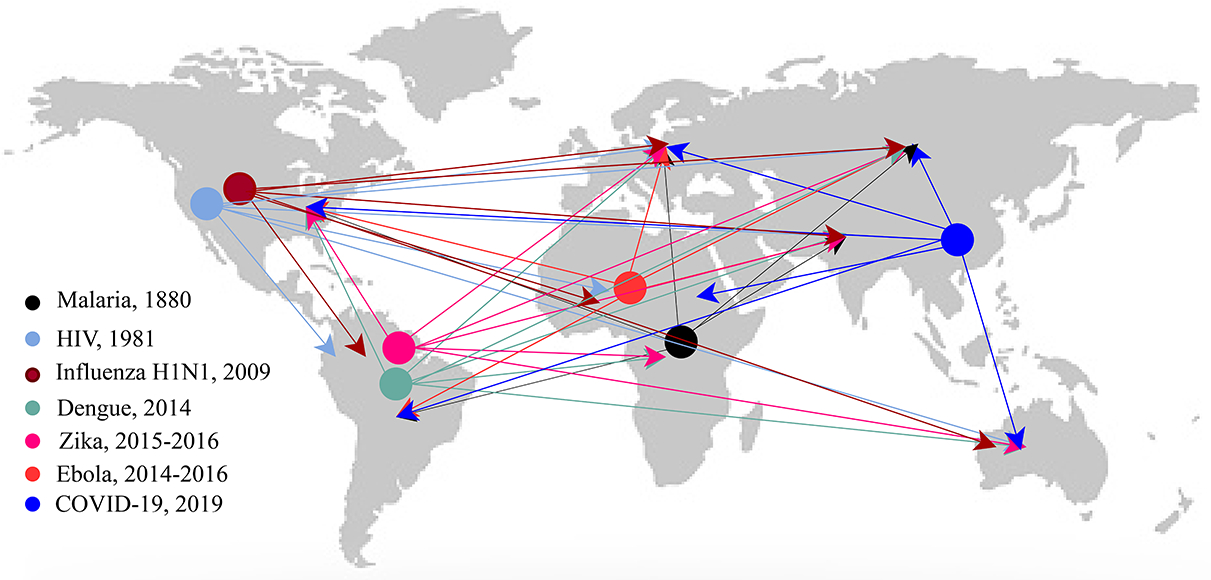
Illustrates the outbreak of each disease and its spread via human movement to other places. Seven types of infectious diseases are presented in this figure, and there has not been any outbreak of hepatitis in recent decades (Cox 2010, De Cock, Jaffe, and Curran 2012, Patel et al. 2010).

Before delving into the discussion regarding the chosen articles for review, we illustrate the transmission of infectious diseases via human mobility in Figure 3. The figure illustrates the spread of selected infectious disease outbreaks (De Cock, Jaffe, and Curran 2012, Cox 2010, Patel et al. 2010), although it is important to note that it does not necessarily indicate the origin of the virus as there may be some controversy regarding the starting location of certain diseases due to the lack of solid evidence.

### 3.2. Vector-borne diseases

**Figure 4.**
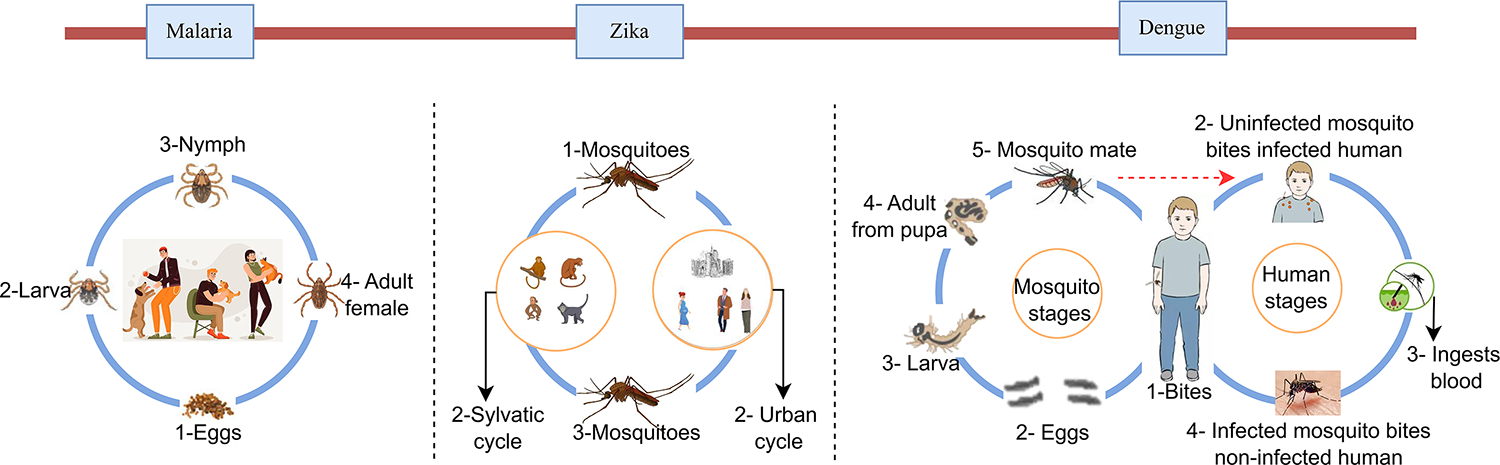
The life cycle of malaria, Zika, and dengue viruses is depicted for enhanced comprehension.

Vector-borne diseases (VBD) caused by parasites, bacteria, and viruses are transmitted to humans by vectors (Figure 4). Each year, there are over 700,000 deaths from vector-borne diseases, such as malaria, dengue, Zika, yellow fever, and other VBDs. The burden of such diseases is disproportionately high in tropical and subtropical regions and excessively affects the poorest people. In recent years, major outbreaks of Zika, dengue, and malaria have afflicted populations in various regions of the world and claimed many lives. The virus is transmitted by the bite of infected mosquitoes to humans, and then humans spread the disease via movement into new areas (Wilson et al. 2020). In this category of viruses, Zika, dengue, and malaria have garnered significant attention from researchers, while other vector-borne diseases (VBDs) have received comparatively less focus. Subsequently, few studies have explored the association between the spread of such viruses and human mobility. This disparity is evident in our literature search, as we were able to retrieve more than ten relevant articles solely for Zika, dengue, and malaria based on the defined search terms.

#### 3.2.1. Zika virus

Zika virus is a mosquito-borne disease that was first isolated in 1947 from the Zika forest in Uganda (Dick, Stuart F, and Alexander 1952). Since its inception, several outbreaks have been recorded in various parts of the world. The primary mode of transmission for this disease is through the bite of Aedes spp. mosquitoes. However, the virus can also be transmitted via sexual practices, blood transfusions, and from mother to fetus during pregnancy. Of particular concern, infants whose mothers contract the virus during pregnancy may be born with microcephaly, a serious condition that affects brain development.

Over decades of studies, many precautionary measures are regarded to be effective in controlling the Zika virus, including the human mobility factor (Wesolowski et al. 2016); moving within and between regions with Zika virus increases the probability of carrying the virus between places by the infected individuals. The Zika virus outbreak between 2015 and 2016 in America prompted the World Health Organization to announce an emergency situation. Numerous studies have explored the impact of human mobility on the transmission of the Zika virus, considering a variety of factors. For example, some studies aimed to identify which demographic groups are more likely to travel to affected areas (Teich et al. 2018) and how the virus has influenced travelers’ plans (Widmar et al. 2017). Other studies have assessed public awareness of the Zika virus, especially among pregnant women during their travels (Guo et al. 2017, Squiers et al. 2018), as well as the perceptions of pregnant women in Zika-affected regions regarding travel and mobility (Chandrasekaran et al. 2017). Further studies have investigated the importation and exportation of the Zika virus across different regions, such as its importation into Australia by travelers (Pyke et al. 2014, Quam and Wilder-Smith 2016) and imported cases identified at a travel center in Paris (Sokal et al. 2016). One study focused on strategies to minimize the risk of Zika virus outbreaks caused by international travelers (Angelo et al. 2020). Infectious disease prediction based on human mobility is another important area of research aimed at curbing virus outbreaks. For instance, stochastic models utilizing human mobility data were employed to forecast the 2015–2016 Zika virus outbreak in Colombia (Perrotta et al. 2022), emphasizing the significance of human movement in disease outbreaks. Reconstructing past Zika outbreaks and projecting future spread using mobility and demographic data can help public health officials prepare for and prevent future outbreaks (Zhang, Sun, et al. 2017). The reviewed studies largely applied regression models, mathematical models, and stochastic approaches to primarily survey data and occasionally other sources like mobile phone and air travel data, with most research conducted in the Americas.

#### 3.2.2. Dengue virus

Dengue virus (DENV) is another viral infectious disease transmitted to humans through female mosquito bites, and it has rapidly spread in most regions, largely in tropical and subtropical areas. At present, the geographical distribution of DENV includes approximately 141 countries. One study indicated that more than 390 million infections cause by DENV per year, and Asian countries carry over 70% of the actual burden (Bhatt et al. 2013), and nearly 3.9 billion individuals are at risk of infection with DENV globally (Brady et al. 2012). The virus is transmitted to mosquitoes from humans who are viremic with DENV, and vice versa (Duong et al. 2015).

Numerous studies have revealed that human mobility can play an integral role in the dengue virus spark by characterizing people’s potential contact with the infected sources. Human movement noticeably expands the spatial scale of spreading and causes contact to happen throughout an infected individual’s activity space (Schaber et al. 2021). The dengue pathogen is transmitted by invasive mosquitoes associated with the Aedes in various regions. Several studies have investigated the role of environmental factors in the transmission of the dengue virus (DENV). Among these, several found that human mobility, as tracked by mobile phone data, is a critical driver in the spatial spread of the virus (Kiang et al. 2021, Wesolowski et al. 2015). Other research highlighted the importance of understanding mobility patterns and seasonality for predicting dengue outbreaks at the neighborhood level (Bomfim et al. 2020, Heidrich et al. 2021). The influence of human movement on DENV transmission varies depending on the scale of endemic regions, making this knowledge essential for implementing effective control measures (Falcon-Lezama et al. 2017). Additionally, factors such as symptom severity and the scale of infection can impact daily human mobility (Schaber et al. 2019). The extent of human movement and the regions traversed also play crucial roles in the severity of dengue outbreaks (Tsuzuki et al. 2014). Using stochastic models, some studies have demonstrated that human movement may indeed be the primary driving force behind the dynamics of diseases like DENV (Barmak, Dorso, and Otero 2016, Zhu et al. 2019). Social media data has also proven to be a valuable source in studying the spread of the dengue virus. For example, researchers have used this data to predict the intra-urban spread of dengue (Ramadona et al. 2019) and analyze spatiotemporal patterns based on human mobility extracted from geolocated tweets (Kraemer et al. 2018, Castro et al. 2021). In fact, understanding the factors that contribute to transmission is crucial for containing the virus. This includes further investigation into the influence of human mobility patterns on dengue virus outbreaks.

#### 3.2.3. Malaria virus

Malaria is an acute illness caused by parasites that are transmitted to humans via female Anopheles mosquito bites, and It is a preventable and curable disease. African countries carry a disproportionately high percentage of the global malaria burden. Many western nations, however, succeeded in eliminating the disease during the first half of the twentieth century (Greenwood and Theonest 2002).

**Figure 5.**
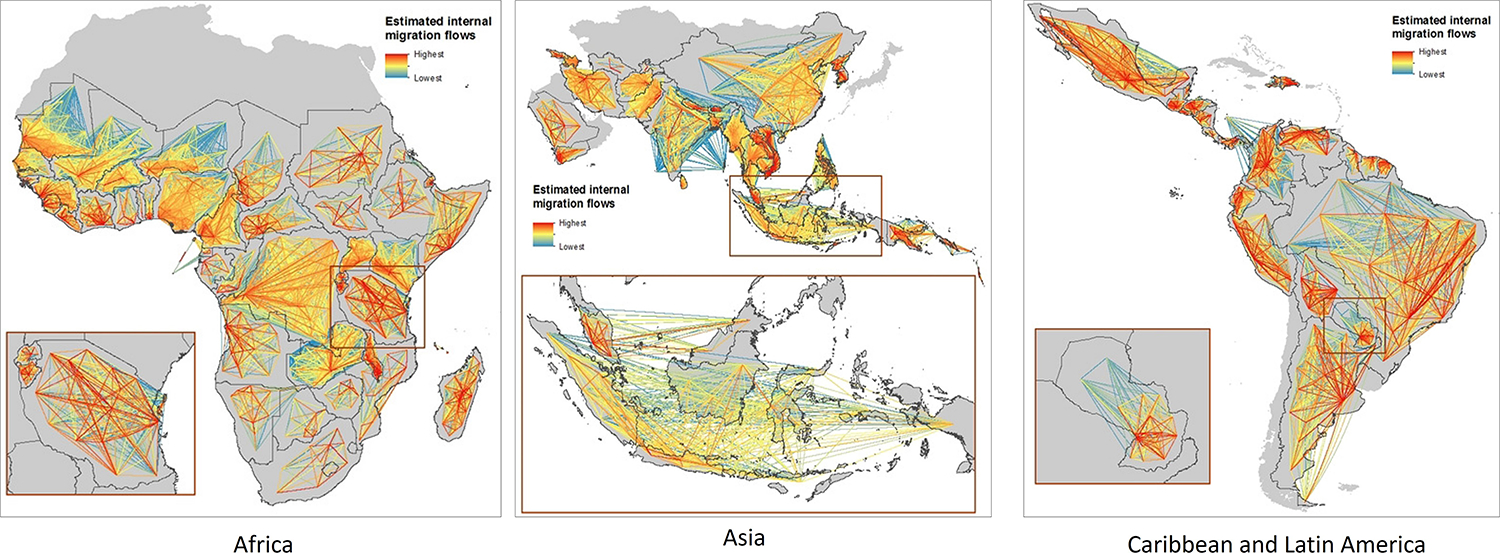
Estimated domestic human migration patterns between subnational administrative divisions of countries where malaria was endemic in 2015 (WorldPop).

Human mobility plays a significant role in the persistence of malaria, even though the disease is not contagious and cannot spread directly from one person to another. Extensive research has been conducted on malaria elimination worldwide, with a focus on human mobility characteristics. For example, studies have investigated how to effectively target mobile and migrant populations at high risk of malaria and implement efficient intervention strategies (Smith et al. 2019, Pongvongsa et al. 2019, Volkman et al. 2020, Khamsiriwatchara et al. 2011). Other researchers have examined the contribution of foreign migrant workers and their mobility patterns on malaria transmission, as well as their risk of contracting the disease (Martins et al. 2020, Hlaing et al. 2015, Schicker et al. 2015, Wai et al. 2014). The connectivity of endemic regions through mobile populations has also been investigated, as understanding how these regions contribute to malaria spread is crucial (Pindolia et al. 2013, Guerra et al. 2019), as Figure 5 depicts domestic migration patterns in 2015, during a period when malaria was considered an endemic disease. Some studies have focused on providing malaria prevention recommendations and precautionary measures for travelers visiting high-risk regions to reduce the global burden of the disease and limit its spread to non-endemic areas (Shellvarajah, Hatz, and Schlagenhauf 2017, Cave et al. 2003). Additionally, one study involved mapping the travel patterns of infected individuals (Sinha et al. 2020), and other studies have examined the contribution of population flow to malaria persistence (Gomes et al. 2020, Das et al. 2022). Moreover, studies have been conducted to identify the environmental risk factors associated with malaria transmission among non-immune travelers (Texier et al. 2013). and identified key traveler groups relevant to malaria transmission (Marshall et al. 2016). In addition to studies examining the association between human movement and malaria transmission, scholars proposed mathematical and simulation models to predict malaria spread with regard to mobility patterns (Li et al. 2022, Chen et al. 2018, Marshall et al. 2018). A number of studies constructed mobility patterns based on Global Positioning System (GPS) data and then investigated malaria disease (Hast et al. 2022, Hast et al. 2019).

Although African and Asian countries carry the highest burden of malaria cases (Talapko et al. 2019), few studies have been conducted in these two regions. Additionally, a large body of studies on malaria in African counties utilized survey data. In contrast, in other regions, cellphone data and GPS logger devices (Hast et al. 2019) have been used in addition to survey data. Incorporating big data (e.g., mobile phones and social media platforms) into research can reduce bias in outcomes, enable real-time data collection, and provide a larger sample size than traditional data collection methods (Garattini et al. 2019).

### 3.3. Ebola virus

Ebola Virus Disease (EVD), also known as Ebola hemorrhagic fever, is a rare infectious disease resulting in a severe and mostly fatal illness in humans. Ebola outbreaks typically begin from a single case of zoonotic transmission, subsequently human-to-human transmission through direct contact or contact with contaminated bodily fluids or infected fomites (Jacob et al. 2020). Among several sources of infection, human-to-human transmission has been considered a major source of Ebola outbreaks worldwide. For example, health-care professionals and workers have frequently been diagnosed with the Ebola virus since they are in close contact with infected individuals and traveling into endemic regions.

Migration of contaminated animals and human are determined as the source of introducing the virus into regions. The Ebola virus has been extensively studied over decades of research based on utilizing human mobility factors on top of other determinants, including developing prediction models, implementing simple and advanced regression models, and examining the effectiveness of mobility control on the disease. Notable studies on this topic include Vogel et al. (2015), who applied stochastic simulation based on cell phone data to monitor movement and travel patterns in West African countries to shed light on Ebola propagation. Additionally, Djiomba Njankou and Nyabadza (2022) considered applying a mathematical model to examine the potential influence of migrants on the Ebola virus dynamic and investigating how mobility causes to circulate of the virus within communities by utilizing Susceptible-Infected-Isolated-Removed (SIIR) model (Rebaza 2021). Valdez et al. (2015) investigated the propagation of the Ebola virus between countries caused by travel and forecasting Ebola epidemic paths (Kramer et al. 2016). Other studies considered regression analysis to explore the association between different forms of human mobility and Ebola transmission. For instance, factors influencing US travelers domestic travel avoidance due to the confirmed cases of the Ebola virus in late 2014 were described (Cahyanto et al. 2016). In response to the 2014 Ebola outbreak, several countries implemented travel bans in areas with an Ebola outbreak in 2014, aiming to limit the spread of the virus to new regions (Poletto et al. 2014, Peak et al. 2018). On the other hand, there were people with relatively high mobility despite the existence of the Ebola virus in 2014–2016 (Fallah et al. 2022). The US Centers for Disease Control and Prevention (CDC) implemented a monitoring program for travelers who visited West Africa during 2014–2016 (Prue et al. 2019), aiming to prevent the spread of Ebola in the US. These studies showed that human mobility regulation played a pivotal role in curbing the spread of the Ebola virus. EVD research, especially in West Africa, has used diverse data like surveys, flight itineraries, and cellphone data to study human movement and virus transmission, but the use of geolocation information from social media platforms for Ebola virus forecasting remains underexplored.

### 3.4. Human Immunodeficiency Virus (HIV)

HIV remains a significant global public health issue, with over 40 million lives lost to the disease so far. The virus can be transmitted through the exchange of body fluids from infected individuals, including semen, vaginal secretions, blood, breast milk, and from mother to child. Although there is no specific cure for HIV infections (Shaw and Eric 2012, Cohen 1998), effective prevention programs, early diagnosis, and access to treatment and care have transformed HIV into a manageable chronic health condition, allowing people to live longer, healthier lives.

Over many years of research, it has been proven that human mobility plays a critical role in the prevention and treatment of HIV. In recent years, the world has faced significant challenges caused by human mobility; the rate of domestic and international displaced populations is at a record high, and pressures caused by migration are increasing, particularly for those who live in the lowest-resource settings. According to the literature review, three possible scenarios can be considered during the migration in terms of HIV spread: (1) migrants travel to destinations from regions with a high risk of HIV infections. In this case, the risk of HIV transmission potentially increases in the host destinations (von Wyl et al. 2011); (2) migrants travel from low-risk regions to high-risk areas of HIV. In this case, migrants are possibly infected and carry the virus to their own regions (Rai et al. 2014), and (3) both the origins and destinations are regarded as high-risk areas (Agadjanian 2016). Factors such as travel conditions, accommodations, and work environments can increase migrants’ risk of exposure to HIV. Separation from their families and spouses may lead a significant portion of migrants to engage in sexual behaviors that increase their risk of contracting HIV (Wang et al. 2013). HIV prevalence varies among different mobile groups. For example, studies show that HIV risk is significantly high among the drivers whose trip is longer and who spend nights on their trip (Delany-Moretlwe et al. 2014), and female sex workers who experienced forced sex and violence (Pannetier et al. 2018). In addition, knowledge regarding HIV and migrants’ perceptions and practices vary across different mobile groups (Agadjanian, Carlos, and Boaventura 2011). Furthermore, some mobile populations may face barriers to accessing HIV prevention and treatment services, including a lack of HIV treatment centers (Vignier et al. 2018). Human mobility facilitates the geographic transmission of HIV infectious disease at the local and global scale. Thus, understanding mobility patterns will greatly improve HIV prevention programs, manage HIV, and decrease the spread of the HIV virus. Predominantly based in Africa and Asia, the selected HIV studies primarily employed various regression models and relied heavily on survey data. Fewer studies considered mathematical and stochastic models or alternative data sources like mobile location and social media geolocation data for forecasting HIV transmission.

### 3.5. Influenza virus

Influenza is an acute respiratory infection and a global threat to human health, and it caused four pandemics in the last century. As the existing knowledge regarding the influenza virus evolution, the transmission and distribution of this virus have increased, and avenues to future pandemics have become apparent (Harrington, Kackos, and Webby 2021). The influenza virus spreads rapidly, particularly in crowded places such as nursing homes and educational centers. This demonstrates the pivotal role human movement plays in transporting the virus to new regions.

When an infected individual coughs or sneezes, infectious droplets are dispersed into the environment and can spread up to a meter; if another person visits the contaminated environment, the individual will likely be infected and move the virus to a new area. Studies confirmed that human mobility is a source of transmission. For instance, during the 2016–2017 influenza season, the proportion of mobility significantly reduced among female populations and individuals with higher socioeconomic conditions in order to minimize the risk of influenza virus spread (Eum and Yoo 2022). The virus can also be transmitted by infected passengers on international flights (Foxwell et al. 2011), and then transmitted to new communities. In 2007, a significant outbreak of influenza occurred in Australia, prompting the government to implement movement restrictions and quarantine for infected people to contain the virus (Taylor et al. 2008). It is also evident that traveling by jet aircraft is a great vector for influenza transmission, and potentially can be reduced if travelers consider precautionary measures (Hanley and Birthe 2010, Masuet-Aumatell, Toovey, and Zuckerman 2013, Yanni et al. 2010). Furthermore, the behaviors of international travelers also play a key role in the transmission of the influenza virus, including attitudes and perceptions of travelers (Sharangpani et al. 2011). More recently, another wave of the influenza epidemic was documented in New South Wales in 2018, with overseas and local travel identified as the primary drivers of the epidemic (Marsh et al. 2022). There is growing evidence to suggest that human mobility plays a role in the transmission of the virus. Yet, only one study indicated that there was little evidence to support the association between influenza and human mobility in Hawaii and the United States-affiliated Pacific Islands (USAPI), in 2009 (Miller et al. 2012). Nevertheless, understanding human mobility patterns is widely regarded as an essential element in efforts to reduce influenza transmission and ultimately eradicate the virus (Wesolowski et al. 2012). The selected influenza studies, largely from the Americas and Australia, commonly used statistical methods and occasionally neural networks for estimating transmission pathways.

### 3.6. Hepatitis virus

Hepatitis is a general term referring to inflammation of the liver and is caused by five main hepatitis viruses: A, B, C, D, and E. Hepatitis A and E are typically caused by the ingestion of contaminated water or food, while the other types mainly result from parenteral contact with contaminated body fluids. Common modes of transmission include receiving infected blood, using contaminated equipment, transmission between family members, through sexual contact, and from mother to baby (WHO 2021).

Studies show that those who travel to hepatitis-endemic countries are at great risk of being infected, which then leads to the spread of the virus in their home country (Lu et al. 2018). Specific guidance is constituted by many developed countries for in- and out travelers who are from hepatitis endemic regions or visiting areas with a high risk of hepatitis virus, and provide awareness programs for these groups of population (Nielsen, Petersen, and Larsen 2009, Nielsen et al. 2012, Frew et al. 2017). For example, in 2009, East Asia and Southeast Asia were considered highly endemic zones for hepatitis B and included many tourist destinations for Australians (Leggat et al. 2009), and travelers were encouraged to take precautionary measures to reduce the possibility of virus transmission back to their home countries. Similarly, travelers, especially those visiting endemic regions, have been identified as a primary cause of hepatitis A and B virus transmission in European countries (Pedersini et al. 2016, Zuckerman and Hoet 2008). Likewise, countries that accept refugees and migrants from war-torn countries have witnessed significant increases in the incidence of hepatitis, as observed in Australia (Sievert et al. 2018). Upon relocation to host countries, these groups often face barriers such as limited access to healthcare services and challenges related to their socioeconomic status (Tiittala et al. 2018, Kowdley et al. 2012). As human mobility has been recognized as a factor in the transmission of hepatitis viruses between countries, regions with a high risk of hepatitis infection may require more stringent monitoring strategies regarding human movement. The literature review reveals a concentration of studies on Europe and Australia, using various regression analyses for methodology similar to other diseases.

### 3.7. Coronavirus disease of 2019 (COVID-19)

COVID-19 is an infectious disease related to the SARS-CoV-2 virus, and it was first reported in Wuhan, China (Li et al. 2020). The global spread of COVID-19 and the thousands of deaths in the early stage of the outbreak caused by COVID-19 led the World Health Organization to announce a pandemic on the 12^th^ of March 2020. The health impact of COVID-19 vary across different groups. Often, infected populations develop mild to moderate illness, and they recover without hospitalization (Ciotti et al. 2020). Coronavirus can be transmitted through direct contact (human-to-human or droplet) and indirect contact (infected objects and airborne contagion). The transmission of COVID-19 has been facilitated by human interactions, which are often associated with movement. Studies have shown that both local and long-distance movements play a significant role in the spread of the virus within and among communities (Alessandretti 2022). To prevent transmission, many countries have implemented restriction policies on human movement (Gao et al. 2020, Her et al. 2022, Li et al. 2020), and global mobility patterns have profoundly changed (Dobbie et al. 2022, Bao et al. 2022, Sigler et al. 2021). Researchers have put substantial efforts into exploring the relationship between human mobility and coronavirus transmission (Lee et al. 2020).

The COVID-19 outbreak stands out as one of the largest pandemics ever recorded in modern human history, and it significantly impacted global human mobility patterns during the pandemic (Sigler et al. 2021). Research indicates that the interconnectedness fostered by globalization significantly expedited the dissemination of COVID-19, despite many countries implemented travel and mobility restrictions. This suggests that the global flow of goods, services, and people inherent to globalization can undermine containment strategies, making the virus more difficult to control (Bickley et al. 2021, Vannoni et al. 2020). Additionally, a study analysed cross-border mobility data revealed that containment measures implemented in the destination country, coupled with school closures in the country of origin, were more successful in reducing cross-border movements than either international travel bans or fears related to COVID-19 case numbers and deaths (Docquier et al. 2022). Nonetheless, while numerous countries enacted early movement restrictions to mitigate the spread of COVID-19, tackling such a global pandemic effectively calls for international collaboration to adopt unified and effective measures (Assefa et al. 2022).

In an effort to decelerate the spread of the virus, the majority of countries constituted movement restrictions, resulting in significant declines in almost all transport modes. Public transport, in particular, experienced the biggest drop in ridership (Hasselwander et al. 2021). Similar research conducted in Australia suggests that public transport ridership has returned but at considerably lower levels compared to pre-pandemic (Currie, Jain, and Aston 2021). The impact of COVID-19 on human mobility varied across different regions, with low-income populations in sub-Saharan Africa have experienced a reduction in travel and accessibility due to the decline of welfare and transport services (Behrens and Newlands 2022). However, the pandemic also offered some individuals the opportunity to explore alternative daily routines that may persist post-COVID-19. As such, transportation planners need to understand these alterations and effectively design future travel scheduling to gain public trust in using public transportation (Dianat, Hawkins, and Habib 2022, Zheng, Luo, and Ritchie 2021). These findings underscore the urgent need to develop a more robust public transportation system that can cope with future infectious disease outbreaks and be more resilient in the face of disruptions.

Studies revealed that the impact of COVID-19 on transportation modes varied significantly. Active modes of transportation, such as motorcycles, biking, and personal cars, were perceived as the least risky mode of transportation during the pandemic (Dingil and Esztergár-Kiss 2021). As walking and biking increased during the pandemic in Spain, the majority of people agreed to increase space for pedestrians and cyclists and recommended more restrictions on vehicle mobility (Awad-Nunez et al. 2021). As personal cars were perceived as a safer mode of transportation, people became more interested in purchasing cars for short trips rather than relying on public transportation (Luan et al. 2021). The pandemic had a particularly significant impact on those who relied on transit services, resulting in many individuals shifting to walking as their primary mode of transportation (Parker et al. 2021). Although transit services were considerably influenced during the early stages of the outbreak, public transportation gradually regained some trust after the easing of lockdowns (Ferreira et al. 2022). In Southeast Asian countries where personal car ownership is limited, public transportation continued to be the primary mode of transportation even during the pandemic, a significant portion of the population utilized non-motorized modes of transportation (Paul et al. 2021, Abdullah et al. 2021). In fact, travelers underwent considerable changes in their transportation modes during the pandemic, as compared to the pre- and post-pandemic periods (Nikiforiadis et al. 2022). Comprehending these changing patterns can assist policymakers in turning the COVID-19 pandemic into an opportunity to invest in sustainable and flexible public transportation infrastructure (Ehsani et al. 2021). By addressing the evolving transportation needs of individuals and communities, countries can ensure that their transportation systems are better prepared to withstand future pandemics and other potential disruptions. The pandemic underscores the significance of having flexible and adaptable transportation systems that can cater to the changing needs of individuals and communities during infectious disease outbreaks.

The COVID-19 pandemic significantly reshaped human mobility from various aspects and shed light on existing societal challenges, including how the impacts of pandemics can vary across diverse population groups based on their socioeconomic status (SES), ethnicity, and age groups (Gauvin et al. 2021, Zeng et al. 2022). Numerous studies have confirmed that people in low SES regions continued to move even during the pandemic, particularly those employed in retail occupations (Wang et al. 2022, Levin et al. 2021, Roman et al. 2021, Brough, Freedman, and Phillips 2021). Similarly, evidence strongly suggests that COVID-19 incidence was unevenly distributed, possibly due to higher mobility rates, particularly among those individuals with lower SES (Lamb, Kandula, and Shaman 2021, Yechezkel et al. 2021). Moreover, mobility data revealed that non-white populations encountered higher exposure risks in the US (Coleman et al. 2022). Researchers also investigated the environmental factors influencing the daily mobility of older adults during COVID-19 and discovered that the duration of active travel significantly decreased for this demographic (from 174.72 to 76.92 minutes per week) (Choe, Du, and Sun 2022). The pandemic’s impacts also varied across gender groups (Mejía-Dorantes, Montero, and Barceló 2021). These findings illustrate that the impacts of infectious disease outbreaks can be different across various population groups in terms of their mobility patterns, with higher mobility observed among less-educated people, lower-income populations, and migrants. These insights highlight the need for policymakers to address these disparities and ensure that transportation systems can meet the needs of all individuals and communities, especially during pandemics. Implementing such policies can assist in ensuring that everyone has access to safe and reliable transportation options, irrespective of their socioeconomic status, ethnicity, age, or gender.

Robust prediction models can effectively forecast the outbreak of the disease based on the availability of mobility data (Li et al. 2021) and other historical data; governments can then implement restriction policies to reduce the transmission of infectious diseases. Over the course of the COVID-19 pandemic, studies used various prediction approaches, for instance, proposing prediction models based on connectedness, mobility patterns, and digital proximity tracing for predicting new cases of COVID-19 (Vahedi, Karimzadeh, and Zoraghein 2021, Cencetti et al. 2021). Moreover, statistical models were deployed to effectively forecast new cases based on public mobility data (Ilin et al. 2021, Guan et al. 2021, Kuniya 2020) and predict international mobility that allowed the model to detect disease transmission (Luca et al. 2022). Other scholars considered developing mathematical models for simulating COVID-19 transmission and examined how transmission varied over time (Chen et al. 2020, Ivorra et al. 2020, Giordano et al. 2020, Kucharski et al. 2020) and then combined stochastic models with data recorded cases of COVID-19. Furthermore, graph modeling was also deployed, and it was able to identify undetected cases of infections based on mobility patterns and social distancing measures (Alguliyev, Aliguliyev, and Yusifov 2021). Examining international and domestic mobility trends during the COVID-19 pandemic has been crucial for understanding the spread of the virus (Rowe et al. 2023, Shepherd et al. 2021). Identifying regions with a higher risk of infection and analyzing the influence of human mobility on potential future COVID-19 outbreaks can provide valuable insights for policymakers and public health officials (Chang et al. 2021). Similarly, the Long Short Term Memory neural network model was implemented to forecast the rate of new cases, and the model was first trained based on factual data related to virus transmission (Banerjee and Lian 2022). All in all, comprehensive predictive approaches can significantly reduce virus transmission and accurately identify regions with higher vulnerability using mobility data and other clinical data.

## 4. Discussion

Key findings of published articles discussing the association between human mobility and eight types of infectious diseases are summarized in this review paper. In an era of pandemics, mobility can indeed disrupt the efforts to isolate and treat infectious diseases. Early and timely responses are essential to prevent a pandemic crisis. In light of the significant impact of human mobility on the spread of various infectious diseases, many countries have formalized preventive measures for travelers, including travel restrictions during an infectious disease pandemic. For instance, the early implementation of the lockdown in Wuhan considerably decelerated the spread of COVID-19 at the initial stage of the pandemic (Lau et al. 2020). Similarly, mobility restrictions were implemented in Sierra Leone due to an unprecedented scale of Ebola virus cases in 2015 (Peak et al. 2018). In 2009, public health authorities identified East Asia and Southeast Asia as regions with high endemic rates of hepatitis B, which are also popular tourist destinations. As a result, travelers were advised to take preventive measures to reduce the risk of bringing the virus back to their home countries (Sievert et al. 2018, Leggat et al. 2009). Similarly, government health agencies have issued recommendations for travelers to take precautions when visiting areas known for other infectious diseases, such as influenza and vector-borne illnesses (Foxwell et al. 2011, Prue et al. 2019, Shellvarajah, Hatz, and Schlagenhauf 2017, Falcon-Lezama et al. 2017).

Although a considerable body of research suggests that mobile populations are key contributors to the transmission of infectious diseases, some studies challenge this view. For instance, a study indicated that geographical features had a greater impact on the spread of Ebola than human mobility (Kramer et al. 2016). Likewise, the type of mobility, such as rural-rural versus rural-urban, has been shown to have varying effects on the transmission of HIV (Coffee et al. 2005). Additionally, studies have found that mobile populations may even exhibit lower risk behaviors compared to local populations in the context of HIV (Mantell et al. 2011, Camlin et al. 2017). Human mobility was found to have a lesser role in the spread of influenza in specific U.S. regions (Miller et al. 2012). According to the literature reviewed, the relationship between human mobility and the spread of infectious diseases varies depending on the specific illness. For example, mobility was a significant factor in the spread of COVID-19, whereas its influence appears to be less pronounced in the cases of HIV and influenza. These divergent findings suggest that the relationship between human mobility and infectious disease transmission is complex and not universally applicable, warranting a nuanced approach in public health strategies.

Given the complexities and varying evidence surrounding the topic, we next focus on critical gaps that require further exploration to refine our understanding of this topic. Upcoming sections will specifically address issues related to data quality, methodological advancements, the resulting implications for public health, and policy guidance based on the reviewed articles.

### 4.1. Data quality

Data is the main body of research, and its reliability, accuracy, consistency, and completeness must be considered in a study. Numerous studies probing the connection between human mobility and infectious diseases have relied heavily on survey questionnaires. These can be administered through various channels such as paper, online, mobile, or a combination of these methods. Mobile and online surveys are often the most cost-effective, yet their reach may be limited, potentially excluding participants who can only respond through alternate methods. Traditional face-to-face interviews or paper surveys serve as viable alternatives in such scenarios.

Nonetheless, survey-based studies have challenges. For instance, capturing hard-to-reach respondents can be a significant hurdle. Besides, surveys are prone to response and nonresponse errors, their reach may be limited to specific geographical regions, and they often yield a relatively small sample size (Dillman and Dennis K 2001). Health professionals express particular concern about these errors, especially in oral health surveys (Locker 2000). Moreover, some studies rely on pre-existing datasets from previous research, potentially leading to data that is outdated or lacks comprehensive information. Parameters borrowed from studies conducted in different geographical locations might also skew results due to regional disparities (Zhu et al. 2019). The use of varied data collection methods within a single study can result in a sample that is not accurately representative (Squiers et al. 2018). During the early stages of a disease outbreak, data scarcity can hamper the accuracy and effectiveness of public health interventions and policy decisions. Surveys conducted post-surge may fail to provide an accurate picture of the initial spread. Furthermore, inconsistencies between surveillance data and epidemiological reports can complicate accurate assessments and impede the development of effective public health strategies (Perrotta et al. 2022). Most studies are local, focusing on specific geographical regions. None of the selected articles examined the association of mobility and infectious disease based on regional or global survey data, likely due to the prohibitive costs of large-scale surveys. The tendency to use small sample sizes is a significant limitation, often exacerbated when study areas are remote and challenging to access (Li et al. 2022). Therefore, to surmount these challenges, it is crucial to devise comprehensive survey questionnaires that are representative, reliable, and can be accurately administered across diverse geographical regions (Hill 1998).

Apart from COVID-19, a relatively small proportion of literature considered big data (e.g., mobile phones, GPS, social media) to examine the linkage between mobility and infectious disease transmission. Using mobile and GPS data sources considerably increases the sample size within a certain border and enables monitoring the effectiveness of mobility restrictions, contact tracing, and identifying potential sources of spread. However, while these data sources offer many advantages, they are not without drawbacks. Privacy concerns abound, and capturing cross-border travel patterns can prove challenging. For instance, some countries host a large population of undocumented migrants, and possibly they regularly travel in and out of the host countries; yet, this group is probably reluctant to participate, and lack of their participation may be led to bias (Wesolowski et al. 2015). Likewise, GPS data contain certain errors caused by GPS logger devices. Likely, participants forget to carry the logger or allow others to carry the GPS loggers (Hast et al. 2022). Nonetheless, mobile and GPS data enable researchers to study the connection between human mobility and infectious diseases at a larger scale compared to questionnaires and also offer more comprehensive insights into the dynamics between mobility and infectious diseases. However, given the sensitive nature of such data, it is vital to exercise caution in its collection and analysis.

Social media data sources, such as Twitter, Facebook, Instagram, and Weibo, are valuable sources of mobility data, which allows researchers to conduct insightful studies (Souza et al. 2019, Li et al. 2021). Mobility data extracted from social media platforms have been extensively applied in investigating the association between COVID-19 transmission and human movement patterns, especially Twitter, Facebook, and Weibo (Hu et al. 2021). Such rich resources, however, have not been widely utilized in other infectious diseases, including malaria, Ebola, Zika, HIV, and influenza. Analyzing social media data enable scholars to track human behaviors and activities in addition to extracting mobility patterns. Yet, there has been limited attention given to utilizing these data sources in exploring the connection between human mobility and infectious diseases beyond COVID-19. Future studies can consider integrating various data sources, including large-scale survey data, mobile phone data, and social media (e.g., Facebook, Twitter, Instagram, and Weibo), to conduct research at regional and global levels. While the value of data and information sharing for managing infectious diseases is well-acknowledged, recent public health crises have heightened the need for effective cross-border communication, yet there is a lack of empirical research exploring the factors that influence the transfer and utilization of public health data and expertise (Liverani et al. 2018).

### 4.2. Methodological enhancement

Outbreaks of COVID-19 and pandemic flu or other types of infectious diseases have raised concerns regarding how governments utilize modeling to forecast a disease’s course. Models do not solely assist decision-makers in implementing disease control policies and efficiently administering resources but also inform conceptual frameworks for future research on infecious diseases; if designed models are unsound, they possibly do not generate the reliable predictions required to make good decisions (Grassly and Fraser 2008). Various types of models have been established throughout the course of studies, including statistical approaches, mechanistic state-space models (e.g., predictions, stochastic), and simplified arithmetic approaches (Zhang et al. 2022). Statistical analysis, prediction models, and stochastic simulations are mainly applied in the selected articles for this review.

Approximately 80% of the selected articles utilized statistical analyses—ranging from exploratory analysis to simple and complex regression models—to investigate the relationship between human mobility and infectious diseases and evaluate the effectiveness of social restrictions and interactions. While these analyses typically outline patterns and correlations inherent in the data, they often fall short in determining causal relationships. This limitation is particularly pronounced when multiple factors contribute to an outcome, as not all of these factors may be captured by the available data (Chambers and Trevor 2017). Furthermore, the majority of studies did not develop a robust methodology for handling missing data, which can have significant implications for the accuracy and validity of the research findings (Prue et al. 2019, Wai et al. 2014). Missing data can arise from various sources, such as non-response, measurement errors, or data collection challenges. When not properly addressed in the process of analyses, missing data can lead to biased estimates, reduced statistical power, and incorrect conclusions. In contrast, the majority of studies didn’t acknowledge this challenge in their research. It is essential for researchers to acknowledge the presence of missing data in their analyses, adopt suitable techniques to address this issue and assess the potential impact of missing data on the overall findings of their studies. For instance, to achieve a meaningful result from applying a multivariate analysis, the input data must be relatively large, but most studies only considered a small dataset (Pongvongsa et al. 2019, Fonner et al. 2021) while using this model. Hence, considering a proper model based on the dataset requires full attention for the analyses; otherwise, it is challenging to draw a generalized conclusion.

Nearly 10% of the publications included in this review developed mathematical models to predict the spread of infectious diseases. Major limitations of prediction models are to rely on a set of assumptions, like uniform distribution of cases over the course of the study, assuming equal distribution of cases in the entire study area, and setting constant parameters in the experiments (Quam and Wilder-Smith 2016, Kramer et al. 2016). Modeling approaches, particularly those aimed at understanding complex phenomena such as the spread of infectious diseases and the impact of human mobility, often face challenges in incorporating all relevant factors. Two primary reasons for this limitation are computational complexity and data scarcity (Zhu et al. 2019). This potentially can be addressed by leveraging a hybrid approach that combines data-driven machine learning techniques and mechanistic models with simplified, domain-specific models, allowing for efficient integration of available data and reduced computational demands. Adding measures of non-pharmaceutical interventions (e.g., rate of mobility change, social distancing, isolation), as well as pharmaceutical factors, including vaccine availability, accessibility to health care centers, resource availability, and various types of viral mutations, can enhance the performance of the developed models (Djiomba Njankou and Nyabadza 2022). The characteristics of each type of infectious disease need to be thoroughly reviewed first, to guide the model design. In sexually transmitted diseases, for example, the characteristics of mobility (e.g., short or long distance, domestic or cross-border), socio-demographics of mobile populations (e.g., age, gender, education level, income) (Zhang et al. 2020), and the legal migration status (legal or undocumented) play a fundamental role in disease transmission. The incorporation of all of these characteristics into forecasting models could potentially enhance the accuracy of prediction models. However, the majority of these features were not considered in the selected articles in this review. Likewise, for vector-borne diseases such as Zika, malaria, and dengue, one approach is to identify regions with a higher likelihood of infectious transmission using satellite imagery. This imagery can reveal critical environmental factors such as seasonal changes, vegetation cover, and water bodies, which are known to influence the habitats of disease vectors. After identifying these potential high-risk areas, researchers can then integrate this information with mathematical models. This combined approach provides a more comprehensive understanding of disease transmission patterns, allowing for more effective disease control and prevention strategies (Sinha et al. 2020), while less focus has been given to exploring such data sources. Another promising approach could be the integration of multi-source data based on a hybrid of models across disciplines. Additional challenges for prediction models are the computational complexity and storage, particularly if the given data is huge; considering high-performance computing techniques are promising directions to overcome such challenges (Wang et al. 2021). Long-standing infectious diseases continue to pose challenges to global health infrastructure. Thus, it is important to continually refine and enhance these models, as they play a vital role in developing more effective public health strategies and facilitating a better understanding of the complex dynamics underlying infectious disease transmission and human mobility.

### 4.3. Public health implications

This review paper transcends academic discussion to offer actionable insights crucial for improving public health policy and infectious disease control. By identifying key research gaps and vulnerabilities in existing systems, we highlight the urgent need for targeted action. These findings have considerable implications in informing effective disease control interventions and improving public health outcomes. This review serves not just as academic inquiry but as a vital framework for health professionals and healthcare providers. With this context in mind, we will now delve into the distinct public health ramifications identified by our review.

First, the impact of mobility on infectious diseases varies based on the characteristics of mobility. Migrants and long-distance travelers often face substantial challenges due to factors such as language barriers, discrimination, difficulties in accessing healthcare services, and uncertain legal status in their host countries (Desgrees-du-Lou et al. 2016). Beyond transporting communicable diseases to their destinations, these groups can also become particularly vulnerable to infection. This vulnerability may arise from their destinations being high-risk areas for infectious diseases or from their marginalization and exclusion within the communities they join. Consequently, it’s essential that public health initiatives account for these unique challenges and vulnerabilities to ensure effective disease prevention and control among these groups (Coffee et al. 2005). To successfully promote prevention measures and enhance timely treatment, healthcare services require to recognize this critical challenge and must consider the unique challenges faced by these groups, ensuring that they have access to appropriate healthcare services and preventative measures to protect both themselves and the communities in which they reside. Second, the public plays an essential role in containing a communicable disease if they are well informed regarding infectious disease threats. However, the reviewed articles suggest that public awareness is significantly low among mobile populations regarding infectious diseases (Teich et al. 2018, Guo et al. 2017). Similarly, public perception is critically important toward infectious diseases to decelerate transmission. For instance, the Ebola outbreak in early 2013 rapidly spread beyond the original geographical location due to the lack of precautionary measures by public in the infected communities (Coltart et al. 2017). Therefore, it is considerably important for public health professionals to pay more attention and invest in awareness programs. Third, massive global travel, urbanization, and population growth (Bloom and Cadarette 2019) have intensified the need for more comprehensive research in this area. To address these concerns, public health professionals require to closely conduct research with transportation sectors and implementing effective surveillance systems. Likewise, urban planning and public health intervention must be closely integrated, with an emphasis on creating healthy living environments that minimize the risk of disease transmission while ensuring that healthcare services are accessible and effective for all residents. Subsequently, through interdisciplinary collaboration, researchers can develop more effective strategies to mitigate the spread of infectious diseases. Finally, in an era marked by rapid globalization, both domestic and international travel have increased considerably, profoundly impacting the spread of diseases on local and global scales. This underscores the necessity for health professionals to broaden their research scope beyond local contexts. Such wide-ranging investigations could shed light on how globalization exacerbates global health challenges and help pinpoint why certain regions struggle to stave off the threat of infectious diseases. For instance, African nations bear an excessively high percentage of the global malaria burden, while Asian countries shoulder over 70% of the global dengue virus burden (Bhatt et al. 2013). Understanding the factors contributing to these disparities is vital for crafting effective public health strategies and interventions.

### 4.4. Policy amendment

Recent years have witnessed outbreaks of previously unknown infectious diseases and the rapid spread of known infectious diseases beyond geographic regions where they initially emerged and typically exist, including re-emerging diseases that significantly declined before. More recently COVID-19 pandemic (Wu, Chen, and Chan 2020), despite significant scientific and medical advancements, the global impact of the virus underscores the ongoing risk of outbreaks escalating into future epidemics and pandemics (Coltart et al. 2017, Ciotti et al. 2019). The next COVID-19-like pandemic could hit any time in the future now; it is, therefore, critical that countries establish a worldwide policy to rapidly and effectively respond to any future pandemics before it disrupts health systems and the economy. Policy interventions, in fact, have been proven to be effective in containing diseases and lowering the spread of viruses (van de Pas et al. 2019, Glover et al. 2020).

Research indicates that countries with higher domestic cases of infectious diseases are less likely to implement travel restriction policies (Smith and D. 2006). Interestingly, countries with a larger proportion of older residents are less inclined to enact travel restrictions; moreover, contrary to expectations, countries with more robust healthcare systems are more likely to put travel restriction policies in place (Bickley et al. 2021). Studies also indicate that while countries with high social globalization are quicker to implement travel restrictions (Cash and Narasimhan 2000), those with effective governments and international political ties are less likely to do so. This presents a policy challenge of balancing international cooperation with national public health goals. Policymakers should thus consider the multifaceted impacts of globalization and human mobility when devising strategies to control infectious diseases, potentially through multilateral agreements that address these complexities. Studies also show that marginalized groups are more at risk of vulnerability in a pandemic (Holder-Dixon et al. 2022). Studies indicate that undocumented migrants are at higher risk for HIV and STDs due to factors like sexual behavior, lack of treatment, forced sex, and socioeconomic status (Pannetier et al. 2018, Swain et al. 2011). Certain groups, such as men who have sex with men, are particularly vulnerable (Vanden Berghe et al. 2013, Ramesh et al. 2014, Garcia et al. 2017, Zhang, Martinez-Donate, et al. 2017). These findings call for urgent policy adjustments in countries with high rates of infectious diseases, including easier access to treatment for migrants and measures to reduce violence against them.

### 4.5. Limitation

This study has a number of limitations that are important to acknowledge. First, the review was confined to two databases, Web of Science and PubMed, which do not encompass all journals. There may be a delay of several months before articles from other indexed journals are added to these databases, potentially excluding recent relevant literature. Secondly, the use of unsupervised machine learning in the initial screening stage may have inadvertently excluded some pertinent articles from the primary search pool. Thirdly, this review was restricted to only original, peer-reviewed papers, excluding high-quality articles that are under review and conference proceedings, potentially introducing a selection bias. Furthermore, the distribution of diseases studied was skewed, with approximately 43% of the reviewed papers focusing on COVID-19 and HIV, while the remaining 57% discussed Zika, Ebola, malaria, dengue, influenza, and hepatitis. This could have influenced the search results and the conclusions drawn from them. Given these limitations, future research should aim for a more expansive scope, encompassing a broader array of infectious diseases and human mobility, and consider other databases for a more comprehensive review.

## 5. Conclusion

This systematic review enriches the existing knowledge in the realms of public health and geography. The forces of globalization, urbanization, and population growth are increasingly interconnecting the world, thereby heightening the potential for the rapid spread of infectious diseases across geographical borders and within densely populated regions. With the continuous evolution of these global trends, it is paramount to create innovative, cross-disciplinary strategies in public health that address the intricate nexus between human mobility, environmental factors, and infectious disease transmission dynamics.

Our review suggests that the relationship between human mobility and the transmission of infectious diseases is intricate and not universally applicable. The impact of human mobility exhibits spatial and temporal disparities: spatially, it holds more significance in urban areas compared to rural ones, in regions with higher connectivity as opposed to those with lower connectivity, and in certain geographic areas over others; temporally, it manifests more prominently during the initial stages of outbreaks rather than the later phases. Furthermore, its influence varies across different types of diseases – it is more pronounced in respiratory infectious diseases than in sexually transmitted ones.

Despite an extensive body of research, certain knowledge gaps persist. We have identified four areas that warrant further exploration to deepen our understanding of this field. Research gaps in the existing studies of human mobility and infectious diseases include a limited focus on diseases other than COVID-19 and HIV, reliance on survey data with its inherent limitations, a predominant use of statistical analyses over mathematical modeling, and a local rather than global perspective. These gaps hinder our understanding and the development of comprehensive strategies. Furthermore, this review highlights critical gaps in public health policy and infectious disease control, emphasizing the unique vulnerabilities of migrants and long-distance travelers. It underscores the need for tailored public health initiatives and better public awareness programs, particularly for mobile populations. The review also calls for interdisciplinary collaboration to address the challenges of globalization, urbanization, and varying disease burdens across regions. These actionable insights aim to guide public health professionals in crafting more effective strategies for improved health outcomes.

Additionally, in this era, despite scientific and medical advancements, the threat of infectious disease outbreaks evolving into global pandemics remains. Countries exhibit various approaches to public health measures like travel restrictions, often influenced by factors such as domestic case rates, age demographics, healthcare capacity, and international political engagements. Policymakers face the challenge of balancing international cooperation with national public health imperatives. Special attention needs to be given to vulnerable populations, who are at higher risk of infection due to various socio-economic factors and lack of accessibility to medical facilities. The findings underscore the need for a coordinated, multifaceted, and globally minded approach to public health policy to better prepare for and manage future outbreaks.

By addressing these identified areas of knowledge gaps and embracing a holistic, interdisciplinary approach, future research can substantially enhance our comprehension of the complex interplay between human mobility and infectious diseases. This, in turn, will guide the formulation of more precise and effective public health strategies to mitigate the impacts of these diseases, catering to the needs of various populations across different geographical regions.

## Figures and Tables

**Table 1.** Summary of studies related to human mobility and vector-borne diseases.

Authors	Purposes	Methods	Country/Regions	Findings	Disease

(Teich et al. 2018)	Assess awareness among potential travelers to risky areas	Simple statistical analysis	America	Women had a higher level of knowledge than male about zika virus.	Zika
(Guo et al. 2017)	Assess knowledge about Zika among pregnant women	Multivariate logistic regression	America	Their babies’ health was more concerned to them than themselves. About 14% canceled their travel.	Zika
(Perrotta et al. 2022)	Predict Zika virus outbreak from 2015–2016 based on mobility data	Mathematical model	Colombia	Using mobile phone data can improve prediction accuracy	Zika
(Zhang, Sun, et al. 2017)	Simulate the epidemic of Zika virus for several countries in the Americas	Stochastic epidemic model	Americas	The model estimated the spatiotemporal distribution of the Zika virus	Zika
(Angelo et al. 2020)	Contribution of international travelers to Zika transmission	Statistical analysis	Americas and Caribbean	Early Zika viruses were reported in Southeast Asia and Oceania. Then travelers in the Americas and the Caribbean reported infection in 2015.	Zika
(Quam and Wilder-Smith 2016)	Estimate global exportation of Zika virus from Brazil	Mathematical model	Brazil	Estimated that 584–1786 cases were exported from Brazil to other countries in the first year (2014–2015)	Zika
(Zhu et al. 2019)	Human mobility, mosquito control, and temperature on dengue transmission	Mathematical model	China	Mobility and suitable temperature could be leading factors for the transmission of dengue in the Pearl River Delta.	Dengue
(Barmak, Dorso, and Otero 2016)	Estimate dengue virus spread based on mobility data	Stochastic model	Argentina	Human movement could be the driving force for vector borne epidemics	Dengue
(Kumanan, Sujanitha, and Nadarajah 2019)	The influence of population mobility on dengue virus spread	//	Sri Lanka	Human mobility played a key role in the outbreak of dengue in 2010.	Dengue
(Tsuzuki et al. 2014)	Association between migration and dengue virus transmission	Logistic regression	Vietnam	Migration from rural regions potentially increases the risk of dengue transmission in urban areas.	Dengue
(Heidrich et al. 2021)	Forecasting dengue cases based on seasonality and mobility	Mathematical model	Jakarta	Adding temporal factor significantly improve the performance of prediction models.	Dengue
(Wesolowski et al. 2015)	Examine the influence of mobility on dengue epidemics	Developed a fine-scale dynamic risk map	Pakistan	Environmental factors and population flow played key roles in the dengue epidemic.	Dengue
(Bomfim et al. 2020)	Dengue outbreak prediction at the neighborhood level using mobility data	Artificial neural network and mechanistic models	Brazil	Using human mobility data considerably improves the performance of prediction models.	Dengue
(Desjardins et al. 2020)	Examine the knowledge and attitude of mobile population	Generalized linear models	Colombia	The results suggest that knowledge depends on community, attitude and practice relates to individual	Dengue
(Das et al. 2022)	Impacts of mobility on malaria persistence	Mathematical model and stochastic approach	Zanzibar in Tanzania	Malaria will cease in Zanzibar if mainland Tanzania implements restricted measures on travelers.	Malaria
(Li et al. 2022)	Simulate daily travel patterns to and from work for malaria control	An agent-based model	Myanmar	Forest workers were the most mobile population and also experienced higher malaria exposure in both study areas.	Malaria
(Schicker et al. 2015)	Association of malaria movement with mobility patterns among farm workers.	A venue-based sampling model	Ethiopia	Seasonal migrant workers encountered a direct threat of malaria, and malaria prevalence was high among them. This increased their risk of spreading the parasites in their own homes.	Malaria
(Hast et al. 2022)	Mobility patterns in a region of malaria persistent along the border in Mozambique	Simple statistics	Zimbabwe	Participants were highly mobile year-round. Males had greatly higher mobility compared to females.	Malaria
(Chen et al. 2018)	Association between population flow and malaria vulnerability	Stochastic simulation	China-Myanmar border	A high proportion of the mobile population was at greater risk of malaria exposure.	Malaria
(Chang et al. 2019)	Spatial distribution of malaria using parasite genetic and mobility data	Mathematical model	Bangladesh	The highest transmission was in forest regions due to the regular mobility of forest workers and military personnel.	Malaria
(Arisco, Peterka, and Castro 2021)	Malaria spread in the Northern Brazil in gold mining sites	Pearson’s Chi-Square test of regression analysis	Brazil	Human mobility in the Northern Brazil and other neighboring countries like Venezuela and Guyana is the primary factor for the spread of malaria	Malaria

**Table 2.** Summary of studies pertaining to human mobility and Ebola infectious disease.

Authors	Purposes	Methods	Country/Regions	Findings

(Li 2017)	Predict the basic number of cases	Mathematical model	Sierra Leone	It revealed that human movement could play a key role in the outbreak of the virus.
(Rebaza 2021)	Studying transmission dynamics of Ebola in communities	Used matrix and graph-theorem techniques	General	The transmission of the Ebola virus is caused by infected people, who are mostly moving into communities without restrictive measures.
(Poletto et al. 2014)	The impacts of traffic reduction caused by the Ebola outbreak	Numerical simulation	West Africa	Travel bans only delayed the spread of the Ebola virus in West Africa for a limited period.
(Peak et al. 2018)	Impacts of lockdown on mobility during the Ebola outbreak in Sierra Leone	Retrospective analysis of mobile phone records	Sierra Leone	A 3-day lockdown was implemented; there was a 76% trip reduction during the lockdown if the trip was longer than 30 km.
(Kramer et al. 2016)	Predicting the expansion of the Ebola epidemic	Generalized gravity model	Guinea, Liberia, & Sierra Leone	Geographical features strongly affected the spread of Ebola. However, mobility was a poor predictor in the spatial spread model.
(Valdez et al. 2015)	Ebola spread among individual mobile in Liberia	Stochastic and deterministic models	Liberia	The study found that the propagation of the virus was caused directly by travelers among counties. To curb the virus spread, it was suggested to take preventive measures on travelers.

**Table 3.** A brief summary of studies related to HIV and human mobility.

Authors	Purposes	Methods	Country/Regions	Findings

(Camlin et al. 2019)	How mobile individuals are associated with HIV cases	Descriptive statistical analysis	Uganda and Kenya	A higher percentage of male adults compared to females migrated locally. Labor-related migration was greatly associated with HIV cases for both women and men.
(Coffee et al. 2005)	The contribution of rural-urban migration on HIV infection	Multivariate logistic regression	Zimbabwe	The association between mobility and the risk of AIDS infection was not confirmed among rural-urban migrants. Instead, rural-rural migration might cause HIV infection in this study area
(Ramesh et al. 2014)	Mobility and HIV infection among men who have sex with men (MSM)	Multivariate logistic regression	India	26% of MSM were mobile, and mobility was noticeably associated with HIV cases. Mobile individuals had sex at their destination.
(Norris et al. 2017)	Correlation between agricultural plantation migrants and the prevalence of STIs	Multivariate Poisson regression	Tanzania	HIV prevalence was 9% among migrants and 6% among non-migrants. Herpes prevalence was 57% and 32% for migrants and non-migrants, respectively.
(Mantell et al. 2011)	HIV risk imposed by migrants among workers in an entertainment center	Logistic regression	China	Migrants didn’t have higher sexual risk behavior than locals. Indeed, adaptation to local traditions may increase HIV risk behavior over time.
(Reed et al. 2012)	Relationship between high mobility and HIV risk among female sex workers (FSWs)	Logistic and linear regression models	India	About 12% of FSWs were highly mobile, and reported higher HIV risk factors, including physical violence, sexual assault, unprotected sex, and involvement in anal sex.
(Rai et al. 2014)	How migrant workers connect areas with high and low HIV infection by mobility	Logistic regression	India	About 20% of labor migrants substantiated the hypothesis that their mobility link areas of differing HIV infection rates. This group can act as a potential source of HIV at the origin place.
(Camlin et al. 2017)	The degree of mobility and HIV outbreak among female sex workers	Logistic regression model	Kenya	Mobility association was insignificant with HIV prevalence, and a great correlation existed between short-term mobility and multiple sex partners.
(Ikeda et al. 2014)	To assess the impacts of male migration on their female partners in terms of STI	Chi-square test and logistic regression	Guatemala	Lack of adequate education about STIs and having multiple sex partners increased STIs transmission. Also, affiliation to a certain ethnic group was associated with transmission.
(Xiao, Liu, and Wu 2020)	How being single and migration increased HIV transmission through trade sex	Univariate and multivariate analysis	China	Unmarried migrants were at higher HIV risk due to experiencing commercial sex and unsafe sex (21.65% and 50.90%), respectively.
(Gareau and Phillips 2022)	Sexual behaviors of young adult travelers in and outside of Canada	Descriptive analysis	Canada	Sexual activity was less abroad compared to at-home. Nevertheless, alcohol consumption increased, and condom use was reduced while traveling.
(Wei et al. 2014)	Whether migrants’ wives play any role in HIV prevalence	Simple statistical analysis	China	Women whose husbands migrated were at a higher risk of HIV infection than those whose spouses didn’t migrate.

**Table 4.** Summary of Influenza virus and human mobility patterns.

Authors	Purposes	Methods	Country/Regions	Findings

(Miller et al. 2012)	Examine the association between travelers and influenza	Statistical analysis	Hawaii, America	Despite considerable travel, there was no association between travel and influenza transmission.
(Eum and Yoo 2022)	Analyzing the linkage between mobility and influenza spread	Time-stratified case-crossover analysis	America	Mobility reduction is associated with influenza-like symptoms, particularly for females and higher-income individuals.
(Foxwell et al. 2011)	Impacts of international flights on influenza onset in 2009	A retrospective cohort study was designed	Australia	International flights were considered a key driver of influenza spread.
(Chen et al. 2021)	Estimate influenza pathways based on mobility data	Neural network	Japan	The virus didn’t spread to regions with undeveloped road networks.
(Masuet-Aumatell, Toovey, and Zuckerman 2013)	Reducing influenza spread via travelers by attending health centers	A cross-sectional study was designed	Britain	Taking the influenza vaccine significantly reduces the risk of influenza spread for older travelers.
(Yanni et al. 2010)	Understanding attitudes and safety practices of travelers to endemic areas	Multivariate logistic regression	American travelers	The majority of travelers were aware of influenza prevention measures, but 41% took the vaccine, and 43% sought health advice.
(Sharangpani et al. 2011)	Traveler’s behaviors toward the influenza pandemic	Simple statistical analysis	America	Travelers who experienced the disease were willing to visit the physician. Age and race also played a key role in safety measures.
(Marsh et al. 2022)	Association between overseas and domestic travels	Multivariate logistic regression	Australia	Travel was considered the early driver of the influenza epidemic in 2018.

**Table 5.** Summary of studies pertaining to Hepatitis virus and human mobility.

Authors	Purposes	Methods	Country/Regions	Findings

(Lu et al. 2018)	Safety measures among adult travelers to hepatitis-endemic countries	Multivariable logistic regression	America	Although the rate of travel to endemic regions was high, vaccine coverage was low among this group.
(Nielsen, Petersen, and Larsen 2009)	Immunization coverage among travelers	Statistical analysis	Denmark	The level of their information regarding hepatitis was low.
(Nielsen et al. 2012)	Evaluate journey length and other factors related to hepatitis virus transmission	Statistical analysis	Denmark	The rate of vaccine uptake among travelers was low. Traveling with friends or alone probably increased risk.
(Zuckerman and Hoet 2008, Pedersini et al. 2016)	Understanding immunization in travelers	Multivariant regression analysis	European travelers	About 51% traveled to risky areas and didn’t seek health advice. About 15% received the vaccination.
(Leggat et al. 2009)	Precautionary measures among travelers to Southeast and East Asia	Regression analysis	Australia	About 54% sought health advice and were vaccinated. Overall, vaccination coverage was low among them.
(Frew et al. 2017)	Knowledge and practices among travelers and tourists	Binary logistic regression	Thailand	About 60% were not immunized against hepatitis, and 24% knew about the virus transmission
(Sievert et al. 2018)	Challenges of accessing hepatitis treatment among migrants.	Statistical analysis	Australia	Migrants who come from war-torn countries face significant barriers.
(Tiittala et al. 2018)	Undetected cases of hepatitis among Kurdish, Russian, and Somali migrants	Multivariable regression analysis	Finland	The undetected cases were among migrants living alone, daily smokers, and having a previous blood-borne disease.

**Table 6.** Summary of studies related to COVID-19 infectious disease and human mobility patterns.

Authors	Purposes	Methods	Country/Regions	Findings

(Zeng et al. 2022)	How mobility and aging accelerate COVID-19 spread	Statistical analysis	America	Population flow contributed to the geospatial disparities at the county level.
(Brough, Freedman, and Phillips 2021)	Socioeconomic disparities in mobility during COVID-19	Regression analysis	America	The percentage of mobility had a lower decline among low-income individuals.
(Choe, Du, and Sun 2022)	Decreasing mobility among older adults and the role of environmental factors	Multilevel linear regression	Hong Kong,China	Duration of travel minimized from 174 to 76 minutes per week. Environmental factors were also impacted.
(Dianat, Hawkins, and Habib 2022)	The impacts of COVID-19 on activity travel.	Statistical analysis	Canada	The frequency and the mode of transportation changed considerably.
(Currie, Jain, and Aston 2021)	The influence of COVID-19 on transportation modes	Statistical analysis	Australia	The study indicated that travel behavior might be different in post-pandemic than pre-pandemic.
(Behrens and Newlands 2022)	How future travel impacted by COVID-19	Regression analysis	Sub-Saharan Africa	The main impact might be on lower-income individuals and a decline to access to public transportation.
(Awad-Nunez et al. 2021)	Individuals’ accessibility to urban mobility	Binary logit model	Spain	Respondents were more willing to recommend sustainable and resilient transit service.
(Hasselwander et al. 2021)	The impacts of public transportation on mega cities	Statistical analysis	Philippines	A significant decline was observed for all transportation modes. Transit services were unable to fulfill the demand.
(Paul et al. 2021)	The impacts of COVID-19 on mobility patterns in developing countries	Ordinal logistic regression	Bangladesh	Despite public transport reduction, around 9% of people still used buses during the lockdown.
(Ehsani et al. 2021)	mobility patterns pre-, during, and post-pandemic	Statistical analysis	America	Biking didn’t decrease during the COVID-19 pandemic, and it was anticipated to increase considerably.

## Data Availability

Data sharing is not applicable to this article as no new data were created or analyzed in this study.
